# Supplementary material for: Modeling glioblastoma heterogeneity as a dynamic network of cell states
Source: Mol Syst Biol. 2021 Sep 16;17(9):e10105. doi: 10.15252/msb.202010105 (PMC8444284; doi:10.15252/msb.202010105)
Supplement: Supplementary file 6 — Source Data for Figure 5 [file MSB-17-e10105-s004.zip › Figure5A_sourcedata/GSEA_3071/nosigdb_stateV.GseaPreranked.1622795159954/DOROSZKO_U3013MG_CLUSTER_2.html]

Details for gene set DOROSZKO\_U3013MG\_CLUSTER\_2[GSEA]

|  || Dataset | state43071 |
| Phenotype | NoPhenotypeAvailable |
| Upregulated in class | na\_pos |
| GeneSet | DOROSZKO\_U3013MG\_CLUSTER\_2 |
| Enrichment Score (ES) | 0.74953043 |
| Normalized Enrichment Score (NES) | 4.501411 |
| Nominal p-value | 0.0 |
| FDR q-value | 0.0 |
| FWER p-Value | 0.0 |
Table: GSEA Results Summary

  

Fig 1: Enrichment plot: DOROSZKO\_U3013MG\_CLUSTER\_2      
 Profile of the Running ES Score & Positions of GeneSet Members on the Rank Ordered List

  

| PROBE | GENE SYMBOL | GENE\_TITLE | RANK IN GENE LIST | RANK METRIC SCORE | RUNNING ES | CORE ENRICHMENT || 1 | HIST1H4C |  |  | 1 | 2.442 | 0.0122 | Yes |
| 2 | ATAD2 |  |  | 2 | 2.214 | 0.0235 | Yes |
| 3 | ESCO2 |  |  | 3 | 2.193 | 0.0347 | Yes |
| 4 | HMGB2 |  |  | 5 | 2.011 | 0.0447 | Yes |
| 5 | NUSAP1 |  |  | 6 | 2.001 | 0.0549 | Yes |
| 6 | CCP110 |  |  | 9 | 1.920 | 0.0643 | Yes |
| 7 | TOP2A |  |  | 14 | 1.842 | 0.0729 | Yes |
| 8 | DHFR |  |  | 18 | 1.795 | 0.0814 | Yes |
| 9 | C21orf58 |  |  | 20 | 1.772 | 0.0902 | Yes |
| 10 | NCAPG |  |  | 21 | 1.760 | 0.0992 | Yes |
| 11 | RAD51AP1 |  |  | 22 | 1.757 | 0.1081 | Yes |
| 12 | FAM111A |  |  | 24 | 1.738 | 0.1168 | Yes |
| 13 | H2AFX |  |  | 26 | 1.725 | 0.1254 | Yes |
| 14 | PCLAF |  |  | 27 | 1.720 | 0.1341 | Yes |
| 15 | CENPF |  |  | 28 | 1.705 | 0.1428 | Yes |
| 16 | TYMS |  |  | 30 | 1.683 | 0.1511 | Yes |
| 17 | RRM2 |  |  | 34 | 1.660 | 0.1590 | Yes |
| 18 | MYBL2 |  |  | 35 | 1.651 | 0.1674 | Yes |
| 19 | CENPU |  |  | 36 | 1.645 | 0.1758 | Yes |
| 20 | KNL1 |  |  | 38 | 1.629 | 0.1839 | Yes |
| 21 | SGO1 |  |  | 40 | 1.614 | 0.1919 | Yes |
| 22 | MKI67 |  |  | 45 | 1.598 | 0.1992 | Yes |
| 23 | BRCA1 |  |  | 46 | 1.592 | 0.2073 | Yes |
| 24 | NEURL1B |  |  | 51 | 1.571 | 0.2145 | Yes |
| 25 | KIF15 |  |  | 52 | 1.568 | 0.2225 | Yes |
| 26 | FOXM1 |  |  | 53 | 1.550 | 0.2304 | Yes |
| 27 | PBK |  |  | 54 | 1.536 | 0.2382 | Yes |
| 28 | EZH2 |  |  | 55 | 1.534 | 0.2460 | Yes |
| 29 | MXD3 |  |  | 62 | 1.500 | 0.2525 | Yes |
| 30 | GTSE1 |  |  | 66 | 1.491 | 0.2595 | Yes |
| 31 | CDK1 |  |  | 69 | 1.488 | 0.2667 | Yes |
| 32 | SMC2 |  |  | 73 | 1.466 | 0.2735 | Yes |
| 33 | SPC25 |  |  | 76 | 1.442 | 0.2805 | Yes |
| 34 | UBE2T |  |  | 79 | 1.432 | 0.2874 | Yes |
| 35 | CLSPN |  |  | 84 | 1.417 | 0.2938 | Yes |
| 36 | CIP2A |  |  | 86 | 1.410 | 0.3008 | Yes |
| 37 | CDCA2 |  |  | 96 | 1.382 | 0.3061 | Yes |
| 38 | RRM1 |  |  | 99 | 1.364 | 0.3126 | Yes |
| 39 | KIFC1 |  |  | 113 | 1.338 | 0.3169 | Yes |
| 40 | CENPM |  |  | 114 | 1.337 | 0.3237 | Yes |
| 41 | TACC3 |  |  | 116 | 1.334 | 0.3303 | Yes |
| 42 | CDCA5 |  |  | 123 | 1.326 | 0.3358 | Yes |
| 43 | NSD2 |  |  | 124 | 1.325 | 0.3426 | Yes |
| 44 | SMC4 |  |  | 127 | 1.320 | 0.3489 | Yes |
| 45 | TMSB15A |  |  | 135 | 1.306 | 0.3542 | Yes |
| 46 | FEN1 |  |  | 139 | 1.296 | 0.3602 | Yes |
| 47 | RTKN2 |  |  | 143 | 1.285 | 0.3661 | Yes |
| 48 | GMNN |  |  | 146 | 1.280 | 0.3723 | Yes |
| 49 | NCAPD3 |  |  | 155 | 1.261 | 0.3771 | Yes |
| 50 | NCAPD2 |  |  | 157 | 1.252 | 0.3833 | Yes |
| 51 | MIS18BP1 |  |  | 159 | 1.247 | 0.3894 | Yes |
| 52 | TMPO |  |  | 161 | 1.243 | 0.3956 | Yes |
| 53 | CENPK |  |  | 162 | 1.241 | 0.4019 | Yes |
| 54 | CDKN3 |  |  | 163 | 1.239 | 0.4082 | Yes |
| 55 | LMNB1 |  |  | 165 | 1.238 | 0.4143 | Yes |
| 56 | KIF23 |  |  | 167 | 1.235 | 0.4204 | Yes |
| 57 | NCAPH |  |  | 168 | 1.235 | 0.4267 | Yes |
| 58 | CDCA3 |  |  | 169 | 1.230 | 0.4329 | Yes |
| 59 | GGH |  |  | 182 | 1.207 | 0.4367 | Yes |
| 60 | DBF4 |  |  | 189 | 1.197 | 0.4416 | Yes |
| 61 | CDKN2D |  |  | 190 | 1.196 | 0.4477 | Yes |
| 62 | KIF4A |  |  | 191 | 1.193 | 0.4538 | Yes |
| 63 | NUF2 |  |  | 192 | 1.189 | 0.4598 | Yes |
| 64 | FANCI |  |  | 196 | 1.179 | 0.4652 | Yes |
| 65 | USP1 |  |  | 202 | 1.168 | 0.4702 | Yes |
| 66 | ZWINT |  |  | 204 | 1.166 | 0.4759 | Yes |
| 67 | ASPM |  |  | 207 | 1.163 | 0.4815 | Yes |
| 68 | PRC1 |  |  | 211 | 1.161 | 0.4868 | Yes |
| 69 | KIF22 |  |  | 213 | 1.157 | 0.4925 | Yes |
| 70 | UBE2C |  |  | 214 | 1.154 | 0.4984 | Yes |
| 71 | CDKN2C |  |  | 223 | 1.143 | 0.5026 | Yes |
| 72 | BTG3 |  |  | 226 | 1.141 | 0.5080 | Yes |
| 73 | ASF1B |  |  | 237 | 1.130 | 0.5118 | Yes |
| 74 | RFC3 |  |  | 239 | 1.130 | 0.5174 | Yes |
| 75 | NDC80 |  |  | 240 | 1.129 | 0.5231 | Yes |
| 76 | SGO2 |  |  | 244 | 1.126 | 0.5283 | Yes |
| 77 | SOGA1 |  |  | 247 | 1.123 | 0.5336 | Yes |
| 78 | TPX2 |  |  | 248 | 1.122 | 0.5393 | Yes |
| 79 | SAC3D1 |  |  | 252 | 1.118 | 0.5444 | Yes |
| 80 | RAD21 |  |  | 256 | 1.109 | 0.5494 | Yes |
| 81 | KIF14 |  |  | 272 | 1.090 | 0.5520 | Yes |
| 82 | BUB1 |  |  | 277 | 1.084 | 0.5568 | Yes |
| 83 | CKAP2L |  |  | 285 | 1.078 | 0.5609 | Yes |
| 84 | HIRIP3 |  |  | 289 | 1.077 | 0.5658 | Yes |
| 85 | CDK5RAP2 |  |  | 292 | 1.076 | 0.5709 | Yes |
| 86 | ECT2 |  |  | 295 | 1.071 | 0.5759 | Yes |
| 87 | AURKB |  |  | 297 | 1.068 | 0.5812 | Yes |
| 88 | ARHGAP11A |  |  | 303 | 1.064 | 0.5856 | Yes |
| 89 | CCNA2 |  |  | 309 | 1.057 | 0.5900 | Yes |
| 90 | CKAP2 |  |  | 312 | 1.053 | 0.5950 | Yes |
| 91 | PRR11 |  |  | 317 | 1.048 | 0.5995 | Yes |
| 92 | WEE1 |  |  | 322 | 1.044 | 0.6040 | Yes |
| 93 | PIMREG |  |  | 339 | 1.025 | 0.6061 | Yes |
| 94 | KIF11 |  |  | 369 | 0.982 | 0.6054 | Yes |
| 95 | APOLD1 |  |  | 372 | 0.980 | 0.6100 | Yes |
| 96 | DNAJC9 |  |  | 373 | 0.979 | 0.6150 | Yes |
| 97 | PHF19 |  |  | 378 | 0.971 | 0.6192 | Yes |
| 98 | PSMC3IP |  |  | 382 | 0.968 | 0.6235 | Yes |
| 99 | TTK |  |  | 383 | 0.966 | 0.6284 | Yes |
| 100 | RPA3 |  |  | 395 | 0.953 | 0.6311 | Yes |
| 101 | HJURP |  |  | 406 | 0.945 | 0.6340 | Yes |
| 102 | MASTL |  |  | 407 | 0.945 | 0.6388 | Yes |
| 103 | SPC24 |  |  | 414 | 0.938 | 0.6424 | Yes |
| 104 | NUDT1 |  |  | 417 | 0.936 | 0.6468 | Yes |
| 105 | DLGAP5 |  |  | 418 | 0.936 | 0.6515 | Yes |
| 106 | MAD2L1 |  |  | 421 | 0.932 | 0.6559 | Yes |
| 107 | SAP30 |  |  | 424 | 0.930 | 0.6602 | Yes |
| 108 | KPNA2 |  |  | 435 | 0.919 | 0.6629 | Yes |
| 109 | CENPE |  |  | 437 | 0.918 | 0.6674 | Yes |
| 110 | LBR |  |  | 438 | 0.916 | 0.6721 | Yes |
| 111 | RACGAP1 |  |  | 443 | 0.912 | 0.6759 | Yes |
| 112 | FAM83D |  |  | 447 | 0.909 | 0.6800 | Yes |
| 113 | CENPH |  |  | 448 | 0.908 | 0.6846 | Yes |
| 114 | ORC6 |  |  | 449 | 0.908 | 0.6892 | Yes |
| 115 | KNSTRN |  |  | 457 | 0.902 | 0.6924 | Yes |
| 116 | SHCBP1 |  |  | 465 | 0.900 | 0.6956 | Yes |
| 117 | POC1A |  |  | 467 | 0.896 | 0.7000 | Yes |
| 118 | MND1 |  |  | 480 | 0.885 | 0.7021 | Yes |
| 119 | SPAG5 |  |  | 481 | 0.884 | 0.7066 | Yes |
| 120 | PHIP |  |  | 513 | 0.854 | 0.7049 | Yes |
| 121 | UBE2S |  |  | 514 | 0.854 | 0.7093 | Yes |
| 122 | PLK1 |  |  | 526 | 0.850 | 0.7114 | Yes |
| 123 | LMNB2 |  |  | 534 | 0.846 | 0.7144 | Yes |
| 124 | BUB1B |  |  | 535 | 0.846 | 0.7187 | Yes |
| 125 | BIRC5 |  |  | 541 | 0.842 | 0.7220 | Yes |
| 126 | CENPQ |  |  | 557 | 0.825 | 0.7232 | Yes |
| 127 | TEDC1 |  |  | 559 | 0.825 | 0.7272 | Yes |
| 128 | CDC25B |  |  | 566 | 0.820 | 0.7302 | Yes |
| 129 | DIAPH3 |  |  | 585 | 0.808 | 0.7308 | Yes |
| 130 | TK1 |  |  | 591 | 0.806 | 0.7339 | Yes |
| 131 | OIP5 |  |  | 620 | 0.788 | 0.7325 | Yes |
| 132 | HMMR |  |  | 622 | 0.788 | 0.7363 | Yes |
| 133 | CENPA |  |  | 627 | 0.786 | 0.7395 | Yes |
| 134 | SKA3 |  |  | 668 | 0.765 | 0.7356 | Yes |
| 135 | ATAD5 |  |  | 721 | 0.739 | 0.7291 | Yes |
| 136 | ANP32E |  |  | 722 | 0.739 | 0.7329 | Yes |
| 137 | PGP |  |  | 727 | 0.732 | 0.7358 | Yes |
| 138 | CKAP5 |  |  | 752 | 0.723 | 0.7348 | Yes |
| 139 | AURKA |  |  | 761 | 0.718 | 0.7369 | Yes |
| 140 | ACTL6A |  |  | 780 | 0.709 | 0.7370 | Yes |
| 141 | CBX5 |  |  | 783 | 0.708 | 0.7402 | Yes |
| 142 | PSRC1 |  |  | 787 | 0.705 | 0.7432 | Yes |
| 143 | DTYMK |  |  | 800 | 0.700 | 0.7444 | Yes |
| 144 | TRIP13 |  |  | 804 | 0.698 | 0.7474 | Yes |
| 145 | AC026401.3 |  |  | 812 | 0.696 | 0.7495 | Yes |
| 146 | ALYREF |  |  | 847 | 0.680 | 0.7463 | No |
| 147 | BCL7C |  |  | 898 | 0.660 | 0.7399 | No |
| 148 | NEK2 |  |  | 901 | 0.659 | 0.7428 | No |
| 149 | MELK |  |  | 915 | 0.653 | 0.7436 | No |
| 150 | KIF20B |  |  | 934 | 0.647 | 0.7434 | No |
| 151 | PTTG1 |  |  | 936 | 0.647 | 0.7465 | No |
| 152 | CENPN |  |  | 953 | 0.638 | 0.7466 | No |
| 153 | CKS2 |  |  | 966 | 0.634 | 0.7475 | No |
| 154 | MNS1 |  |  | 1034 | 0.613 | 0.7375 | No |
| 155 | CDCA8 |  |  | 1043 | 0.610 | 0.7390 | No |
| 156 | ZWILCH |  |  | 1048 | 0.609 | 0.7413 | No |
| 157 | KIF20A |  |  | 1133 | 0.583 | 0.7278 | No |
| 158 | HMGB3 |  |  | 1154 | 0.577 | 0.7268 | No |
| 159 | DEPDC1 |  |  | 1179 | 0.569 | 0.7250 | No |
| 160 | ITGB3BP |  |  | 1180 | 0.568 | 0.7279 | No |
| 161 | ANP32B |  |  | 1187 | 0.566 | 0.7296 | No |
| 162 | HP1BP3 |  |  | 1198 | 0.562 | 0.7305 | No |
| 163 | PARPBP |  |  | 1210 | 0.560 | 0.7312 | No |
| 164 | CBR3 |  |  | 1221 | 0.556 | 0.7321 | No |
| 165 | EXOSC8 |  |  | 1230 | 0.553 | 0.7333 | No |
| 166 | KIF2C |  |  | 1309 | 0.530 | 0.7208 | No |
| 167 | CCNB2 |  |  | 1373 | 0.513 | 0.7110 | No |
| 168 | SPDL1 |  |  | 1397 | 0.507 | 0.7091 | No |
| 169 | YWHAH |  |  | 1407 | 0.502 | 0.7099 | No |
| 170 | CKLF |  |  | 1480 | 0.486 | 0.6983 | No |
| 171 | CKS1B |  |  | 1502 | 0.479 | 0.6966 | No |
| 172 | CEP55 |  |  | 1508 | 0.479 | 0.6980 | No |
| 173 | LSM8 |  |  | 1524 | 0.476 | 0.6975 | No |
| 174 | POP7 |  |  | 1591 | 0.461 | 0.6869 | No |
| 175 | G2E3 |  |  | 1602 | 0.459 | 0.6873 | No |
| 176 | TUBB4B |  |  | 1659 | 0.444 | 0.6786 | No |
| 177 | CENPW |  |  | 1959 | 0.395 | 0.6220 | No |
| 178 | LSM5 |  |  | 1982 | 0.391 | 0.6197 | No |
| 179 | XPO1 |  |  | 2091 | 0.374 | 0.6005 | No |
| 180 | ANLN |  |  | 2125 | 0.371 | 0.5959 | No |
| 181 | RHEB |  |  | 2192 | 0.363 | 0.5848 | No |
| 182 | SKA2 |  |  | 2445 | 0.329 | 0.5371 | No |
| 183 | CCNB1 |  |  | 2480 | 0.325 | 0.5321 | No |
| 184 | SYPL1 |  |  | 2625 | 0.307 | 0.5054 | No |
| 185 | JPT1 |  |  | 2712 | 0.298 | 0.4901 | No |
| 186 | BUB3 |  |  | 2806 | 0.288 | 0.4733 | No |
| 187 | REEP4 |  |  | 2902 | 0.278 | 0.4561 | No |
| 188 | BCL2L12 |  |  | 2968 | 0.272 | 0.4448 | No |
| 189 | MAGOH |  |  | 3724 | -0.335 | 0.2986 | No |
| 190 | TUBA1C |  |  | 3957 | -0.379 | 0.2550 | No |
| 191 | UACA |  |  | 4621 | -0.604 | 0.1282 | No |
| 192 | MT2A |  |  | 4799 | -0.714 | 0.0972 | No |
Table: GSEA details [plain text format]

  

Fig 2: DOROSZKO\_U3013MG\_CLUSTER\_2: Random ES distribution      
 Gene set null distribution of ES for **DOROSZKO\_U3013MG\_CLUSTER\_2**
